# Bis[(1-methyl-1*H*-benzimidazol-2-yl)methanol-κ^2^ *N* ^3^,*O*]bis­(thio­cyanato-κ*N*)cobalt(II) methanol solvate

**DOI:** 10.1107/S1600536810002114

**Published:** 2010-01-23

**Authors:** Yan-Ling Zhou, Hong Liang, Ming-Hua Zeng

**Affiliations:** a School of Chemistry and Chemical Engineering, Central South University, Changsha 410083, People’s Republic of China; b School of Chemistry and Chemical Engineering, Guangxi Normal University, Guilin 541004, People’s Republic of China

## Abstract

In the mononuclear title complex, [Co(NCS)_2_(C_9_H_10_N_2_O)_2_]·CH_3_OH, the cobalt(II) ion is surrounded by two (1-methyl-1*H*-benzimidazol-2-yl)methanol bidentate ligands and two thio­cyanate ligands, and exhibits a distorted octa­hedral coordination by four N atoms and two O atoms. The structure is consolidated by hydrogen bonds between the organic ligand, thio­cyanate anion and the uncoordinated methanol mol­ecule, leading to a chain along [100].

## Related literature

For the synthesis of the ligand, see: van Albada *et al.* (1995 ▶) and literature cited therein. For the cobalt(II) dithio­cyanato adduct, see: Zeng *et al.* (2006 ▶). For the zinc(II) complex of a similar *N*-heterocycle, see: Zhou *et al.* (2007 ▶).
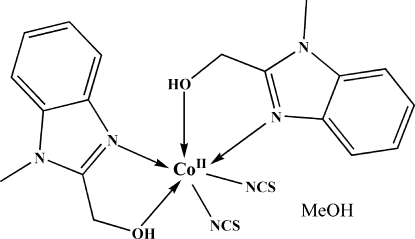


## Experimental

### 

#### Crystal data

[Co(NCS)_2_(C_9_H_10_N_2_O)_2_]·CH_4_O
*M* *_r_* = 531.53
Triclinic, 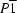

*a* = 7.5008 (13) Å
*b* = 10.3470 (18) Å
*c* = 16.042 (3) Å
α = 95.579 (3)°
β = 103.388 (3)°
γ = 95.179 (3)°
*V* = 1197.3 (4) Å^3^
*Z* = 2
Mo *K*α radiation
μ = 0.93 mm^−1^
*T* = 173 K
0.40 × 0.36 × 0.09 mm

#### Data collection

Bruker SMART APEX CCD area-detector diffractometer
Absorption correction: multi-scan (*SADABS*; Sheldrick, 1996 ▶) *T*_min_ = 0.708, *T*_max_ = 0.921
8542 measured reflections
4146 independent reflections
3288 reflections with *I* > 2σ(*I*)
*R*_int_ = 0.021

#### Refinement

*R*[*F* ^2^ > 2σ(*F* ^2^)] = 0.036
*wR*(*F* ^2^) = 0.116
*S* = 1.01
4146 reflections
298 parameters
H-atom parameters constrained
Δρ_max_ = 0.39 e Å^−3^
Δρ_min_ = −0.26 e Å^−3^

### 

Data collection: *SMART* (Bruker, 2001 ▶); cell refinement: *SAINT* (Bruker, 2001 ▶); data reduction: *SAINT*; program(s) used to solve structure: *SHELXS97* (Sheldrick, 2008 ▶); program(s) used to refine structure: *SHELXL97* (Sheldrick, 2008 ▶); molecular graphics: *X-SEED* (Barbour, 2001 ▶); software used to prepare material for publication: *publCIF* (Westrip, 2010 ▶) and *PLATON* (Spek, 2009 ▶).

## Supplementary Material

Crystal structure: contains datablocks global, I. DOI: 10.1107/S1600536810002114/si2239sup1.cif

Structure factors: contains datablocks I. DOI: 10.1107/S1600536810002114/si2239Isup2.hkl

Additional supplementary materials:  crystallographic information; 3D view; checkCIF report

## Figures and Tables

**Table 1:** Selected bond lengths (Å)

Co1—N6	2.035 (3)
Co1—N5	2.047 (3)
Co1—N1	2.065 (3)
Co1—N3	2.079 (3)
Co1—O1	2.284 (2)
Co1—O2	2.327 (2)

**Table 2:** Hydrogen-bond geometry (Å, °)

*D*—H⋯*A*	*D*—H	H⋯*A*	*D*⋯*A*	*D*—H⋯*A*
O2—H2*A*⋯O3	0.85	1.89	2.689 (3)	155
O3—H3*A*⋯S2^i^	0.85	2.45	3.297 (3)	179
O1—H1⋯S1^i^	0.85	2.36	3.177 (2)	162

Symmetry code: (i) 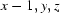.

**Fig. 1.**
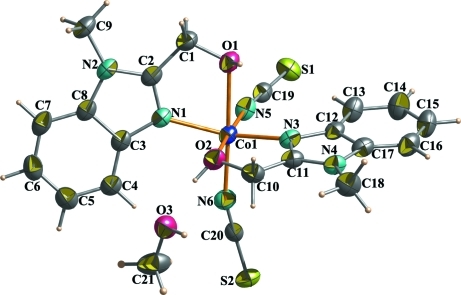
Anisotropic displacement ellipsoid plot of the [Co(II)(NCS)2(C9H10N2O)2] molecule at the 50% probability level; hydrogen atoms are drawn as sphere of arbitrary radius.

**Fig. 2.**
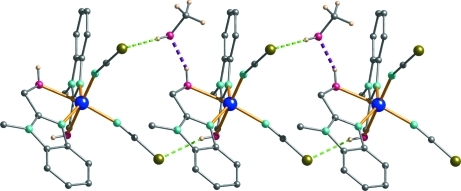
Part of the hydrogen bonded chains along [100] direction. Hydrogen bonds are shown as dashed lines. Symmetry codes: (i) -1 + x, y, z.

## supplementary crystallographic information

### Comment

The benzimidazol alcohols have widely been used as versatile ligands in coordination chemistry, and their metal complexes are of great interest in many fields. Recently, we have reported a few benzimidazol-2-yl methanol base cobalt and zinc complexes (Zeng *et al.* 2006, Zhou *et al.* 2007). In this paper, the title new cobalt(II) complex, (Fig. 1), is reported.

The complex consists of a mononuclear cobalt(II) complex molecule and a methanol molecule. The cobalt(II) ion is surrounded by two [(1-methyl-1*H*-benzimidazol-2-yl)methanol bidentate ligands and two thiocyanato ligands, and exhibits a distorted octahedral coordination by four N atoms and two O atoms (Albada *et al.* 1995) The coordinate bond lengths (Table 1) are typical and comparable to the corresponding values observed in our previously reported similar 2-Hydroxymethylbenzimidazole cobalt(II) complex (Zeng *et al.* 2006).

The structure is consolidated by hydrogen bonds between the organic ligand, thiocyanate anion and the uncoordinated methanol molecule, leading to a one-dimensional chain along the [100] direction. (Table 2, Fig. 2).

### Experimental

(1-methyl-1*H*-benzimidazol-2-yl) methanol was purchased from a chemical supplier. This reagent (0.16 g, 1 mmol), cobalt(II) nitrate hexahydrate (0.15 g, 0.5 mmol) and ammonium thiocyanate(0.08 g, 1 mmol) were dissolved in water (10 ml) that was kept at about 333 K. Red platelets separated from the solution after two weeks.

### Refinement

The C-bound H atoms were placed in calculated positions (C—H = 0.95–0.99 Å) and included in the refinement in the riding-model approximation, with *U*_iso_(H) = 1.2(1.5)*U*_eq_(C,C_methyl_). The hydroxy H atoms were located in a difference Fourier map and refined isotropically with distance restraints of O—H = 0.85 (1) Å, and *U*_iso_(H) = 1.2*U*_eq_(O).

### Crystal data

**Table:**

[Co(NCS)_2_(C_9_H_10_N_2_O)_2_]·CH_4_O	*Z* = 2
*M**_r_* = 531.53	*F*(000) = 550
Triclinic, *P*1	*D*_x_ = 1.474 Mg m^−^^3^
Hall symbol: -P 1	Mo *K*α radiation, λ = 0.71073 Å
*a* = 7.5008 (13) Å	Cell parameters from 4367 reflections
*b* = 10.3470 (18) Å	θ = 2.8–25.0°
*c* = 16.042 (3) Å	µ = 0.93 mm^−^^1^
α = 95.579 (3)°	*T* = 173 K
β = 103.388 (3)°	Plate, red
γ = 95.179 (3)°	0.40 × 0.36 × 0.09 mm
*V* = 1197.3 (4) Å^3^	

### Data collection

**Table:**

Bruker SMART APEX CCD area-detector diffractometer	4146 independent reflections
Radiation source: fine-focus sealed tube	3288 reflections with *I* > 2σ(*I*)
graphite	*R*_int_ = 0.021
phi and ω scans	θ_max_ = 25.0°, θ_min_ = 1.3°
Absorption correction: multi-scan (*SADABS*; Sheldrick, 1996)	*h* = −8→8
*T*_min_ = 0.708, *T*_max_ = 0.921	*k* = −12→12
8542 measured reflections	*l* = −19→19

### Refinement

**Table:**

Refinement on *F*^2^	Primary atom site location: structure-invariant direct methods
Least-squares matrix: full	Secondary atom site location: difference Fourier map
*R*[*F*^2^ > 2σ(*F*^2^)] = 0.036	Hydrogen site location: inferred from neighbouring sites
*wR*(*F*^2^) = 0.116	H-atom parameters constrained
*S* = 1.01	*w* = 1/[σ^2^(*F*_o_^2^) + (0.0643*P*)^2^ + 1.1088*P*] where *P* = (*F*_o_^2^ + 2*F*_c_^2^)/3
4146 reflections	(Δ/σ)_max_ = 0.001
298 parameters	Δρ_max_ = 0.39 e Å^−^^3^
0 restraints	Δρ_min_ = −0.26 e Å^−^^3^

### Special details

**Table:**

Refinement. Refinement of *F*^2^ against ALL reflections. The weighted *R*-factor *wR* and goodness of fit *S* are based on *F*^2^, conventional *R*-factors *R* are based on *F*, with *F* set to zero for negative *F*^2^. The threshold expression of *F*^2^ > σ(*F*^2^) is used only for calculating *R*-factors(gt) *etc*. and is not relevant to the choice of reflections for refinement. *R*-factors based on *F*^2^ are statistically about twice as large as those based on *F*, and *R*- factors based on ALL data will be even larger. In Checkcif report, the following ALERTS were generatedPLAT230_ALERT_2_C Hirshfeld Test Diff for S1–C19.. 6.12 su PLAT230_ALERT_2_C Hirshfeld Test Diff for S2–C20.. 5.57 su PLAT232_ALERT_2_C Hirshfeld Test Diff (M—*X*) Co1–O1..5.19 su Author response: referring to the alert levels C, similar anisotropic displacement ellipsoids were observed in the solvent-free cobalt(II) complex (Zeng *et al.*, 2006), and similar distances for S—C and Co—O (2.268 (2) Å) are found in Zeng *et al.* (2006).

### Fractional atomic coordinates and isotropic or equivalent isotropic displacement parameters (Å^2^)

**Table:**

	*x*	*y*	*z*	*U*_iso_*/*U*_eq_	
Co1	0.49422 (6)	0.79547 (4)	0.71477 (3)	0.03196 (15)	
O1	0.3407 (3)	0.9076 (2)	0.61010 (13)	0.0388 (5)	
H1	0.2553	0.9321	0.6326	0.058*	
O2	0.2200 (3)	0.7225 (2)	0.74875 (14)	0.0420 (6)	
H2A	0.2062	0.6429	0.7575	0.063*	
O3	0.0999 (4)	0.4972 (2)	0.79928 (16)	0.0552 (7)	
H3A	0.0496	0.5315	0.8370	0.083*	
N1	0.3872 (3)	0.6541 (3)	0.61074 (16)	0.0321 (6)	
N2	0.2271 (4)	0.5910 (3)	0.47582 (16)	0.0360 (6)	
N3	0.4426 (3)	0.9434 (3)	0.80037 (16)	0.0335 (6)	
N4	0.3048 (4)	1.0150 (3)	0.90172 (17)	0.0377 (6)	
N5	0.7251 (4)	0.8800 (3)	0.68486 (17)	0.0402 (7)	
N6	0.6311 (4)	0.6910 (3)	0.80523 (19)	0.0451 (7)	
C1	0.2850 (5)	0.8331 (3)	0.5273 (2)	0.0435 (8)	
H1A	0.3651	0.8629	0.4901	0.052*	
H1B	0.1561	0.8449	0.4996	0.052*	
C2	0.2998 (4)	0.6920 (3)	0.53789 (19)	0.0335 (7)	
C3	0.3678 (4)	0.5184 (3)	0.59742 (19)	0.0321 (7)	
C4	0.4280 (4)	0.4266 (3)	0.6519 (2)	0.0365 (7)	
H4A	0.4974	0.4526	0.7096	0.044*	
C5	0.3834 (5)	0.2958 (3)	0.6193 (2)	0.0447 (8)	
H5A	0.4227	0.2310	0.6554	0.054*	
C6	0.2822 (5)	0.2573 (4)	0.5348 (2)	0.0479 (9)	
H6B	0.2539	0.1666	0.5148	0.057*	
C7	0.2218 (5)	0.3459 (4)	0.4795 (2)	0.0430 (8)	
H7A	0.1532	0.3190	0.4218	0.052*	
C8	0.2661 (4)	0.4773 (3)	0.51224 (19)	0.0335 (7)	
C9	0.1212 (5)	0.5970 (4)	0.3872 (2)	0.0444 (8)	
H9A	0.1124	0.6885	0.3776	0.067*	
H9B	−0.0029	0.5510	0.3786	0.067*	
H9C	0.1837	0.5554	0.3462	0.067*	
C10	0.1814 (5)	0.7973 (3)	0.8195 (2)	0.0412 (8)	
H10A	0.1972	0.7475	0.8698	0.049*	
H10B	0.0525	0.8184	0.8045	0.049*	
C11	0.3106 (4)	0.9185 (3)	0.8402 (2)	0.0348 (7)	
C12	0.5277 (4)	1.0679 (3)	0.83793 (19)	0.0332 (7)	
C13	0.6722 (4)	1.1464 (4)	0.8213 (2)	0.0427 (8)	
H13A	0.7323	1.1183	0.7779	0.051*	
C14	0.7252 (5)	1.2669 (4)	0.8701 (2)	0.0508 (9)	
H14A	0.8219	1.3231	0.8589	0.061*	
C15	0.6418 (5)	1.3092 (4)	0.9354 (2)	0.0506 (9)	
H15A	0.6843	1.3923	0.9681	0.061*	
C16	0.4986 (5)	1.2322 (4)	0.9530 (2)	0.0471 (9)	
H16A	0.4411	1.2597	0.9975	0.056*	
C17	0.4430 (4)	1.1128 (3)	0.9026 (2)	0.0373 (7)	
C18	0.1719 (5)	1.0184 (4)	0.9551 (2)	0.0528 (10)	
H18A	0.0893	0.9362	0.9418	0.079*	
H18B	0.0995	1.0916	0.9433	0.079*	
H18C	0.2377	1.0295	1.0162	0.079*	
C19	0.8474 (4)	0.9482 (3)	0.6751 (2)	0.0354 (7)	
C20	0.7421 (4)	0.6650 (3)	0.8619 (2)	0.0317 (7)	
C21	0.2200 (7)	0.4131 (5)	0.8363 (3)	0.0730 (14)	
H21B	0.3474	0.4535	0.8455	0.109*	
H21A	0.2018	0.3309	0.7978	0.109*	
H21C	0.1958	0.3952	0.8918	0.109*	
S2	0.89902 (12)	0.63097 (9)	0.94306 (5)	0.0424 (2)	
S1	1.01807 (12)	1.04930 (9)	0.66347 (6)	0.0445 (2)	

### Atomic displacement parameters (Å^2^)

**Table:**

	*U*^11^	*U*^22^	*U*^33^	*U*^12^	*U*^13^	*U*^23^
Co1	0.0292 (2)	0.0377 (3)	0.0279 (2)	0.00460 (18)	0.00559 (17)	0.00169 (18)
O1	0.0360 (12)	0.0467 (13)	0.0333 (12)	0.0101 (10)	0.0075 (10)	0.0003 (10)
O2	0.0414 (13)	0.0489 (14)	0.0382 (13)	−0.0009 (11)	0.0173 (11)	0.0048 (11)
O3	0.0719 (18)	0.0496 (15)	0.0489 (15)	0.0112 (14)	0.0232 (14)	0.0048 (12)
N1	0.0266 (13)	0.0411 (15)	0.0290 (14)	0.0046 (11)	0.0079 (11)	0.0026 (11)
N2	0.0301 (14)	0.0506 (17)	0.0254 (13)	0.0021 (12)	0.0057 (11)	0.0000 (12)
N3	0.0262 (13)	0.0445 (16)	0.0302 (14)	0.0046 (11)	0.0069 (11)	0.0061 (12)
N4	0.0382 (15)	0.0475 (17)	0.0349 (14)	0.0134 (13)	0.0195 (12)	0.0084 (12)
N5	0.0329 (15)	0.0484 (17)	0.0403 (16)	0.0052 (13)	0.0139 (13)	−0.0018 (13)
N6	0.0438 (17)	0.0477 (18)	0.0396 (16)	0.0114 (14)	−0.0007 (14)	0.0050 (13)
C1	0.044 (2)	0.051 (2)	0.0332 (18)	0.0089 (16)	0.0055 (15)	0.0054 (15)
C2	0.0247 (15)	0.0475 (19)	0.0302 (16)	0.0046 (13)	0.0107 (13)	0.0036 (14)
C3	0.0243 (15)	0.0425 (18)	0.0324 (16)	0.0016 (13)	0.0149 (13)	0.0009 (14)
C4	0.0317 (17)	0.0443 (19)	0.0358 (17)	0.0025 (14)	0.0135 (14)	0.0045 (15)
C5	0.045 (2)	0.043 (2)	0.052 (2)	0.0053 (16)	0.0233 (17)	0.0076 (17)
C6	0.051 (2)	0.040 (2)	0.055 (2)	0.0002 (17)	0.0227 (18)	−0.0050 (17)
C7	0.0381 (19)	0.056 (2)	0.0343 (18)	−0.0029 (16)	0.0159 (15)	−0.0067 (16)
C8	0.0266 (16)	0.0448 (19)	0.0313 (16)	0.0016 (14)	0.0145 (13)	−0.0001 (14)
C9	0.0383 (19)	0.062 (2)	0.0281 (17)	0.0057 (17)	0.0020 (14)	−0.0007 (16)
C10	0.0424 (19)	0.045 (2)	0.0438 (19)	0.0089 (15)	0.0213 (16)	0.0116 (15)
C11	0.0297 (16)	0.0445 (19)	0.0340 (17)	0.0094 (14)	0.0107 (14)	0.0120 (14)
C12	0.0272 (16)	0.0422 (18)	0.0291 (16)	0.0095 (14)	0.0029 (13)	0.0037 (14)
C13	0.0296 (17)	0.053 (2)	0.0430 (19)	0.0019 (15)	0.0110 (15)	−0.0069 (16)
C14	0.0345 (19)	0.058 (2)	0.056 (2)	−0.0017 (17)	0.0112 (17)	−0.0066 (19)
C15	0.041 (2)	0.053 (2)	0.051 (2)	0.0014 (17)	0.0065 (17)	−0.0123 (18)
C16	0.046 (2)	0.059 (2)	0.0378 (19)	0.0163 (18)	0.0111 (16)	−0.0029 (17)
C17	0.0311 (17)	0.050 (2)	0.0332 (17)	0.0120 (15)	0.0091 (14)	0.0066 (15)
C18	0.057 (2)	0.061 (2)	0.054 (2)	0.0158 (19)	0.037 (2)	0.0099 (19)
C19	0.0328 (18)	0.0428 (19)	0.0317 (17)	0.0155 (15)	0.0079 (14)	0.0006 (14)
C20	0.0321 (17)	0.0296 (16)	0.0367 (17)	0.0036 (13)	0.0146 (15)	0.0040 (13)
C21	0.084 (3)	0.101 (4)	0.056 (3)	0.047 (3)	0.039 (2)	0.032 (3)
S2	0.0376 (5)	0.0594 (6)	0.0333 (4)	0.0147 (4)	0.0086 (4)	0.0129 (4)
S1	0.0376 (5)	0.0423 (5)	0.0599 (6)	0.0092 (4)	0.0199 (4)	0.0134 (4)

### Geometric parameters (Å, °)

**Table:**

Co1—N6	2.035 (3)	C5—C6	1.392 (5)
Co1—N5	2.047 (3)	C5—H5A	0.9500
Co1—N1	2.065 (3)	C6—C7	1.371 (5)
Co1—N3	2.079 (3)	C6—H6B	0.9500
Co1—O1	2.284 (2)	C7—C8	1.390 (5)
Co1—O2	2.327 (2)	C7—H7A	0.9500
O1—C1	1.421 (4)	C9—H9A	0.9800
O1—H1	0.8500	C9—H9B	0.9800
O2—C10	1.411 (4)	C9—H9C	0.9800
O2—H2A	0.8500	C10—C11	1.474 (5)
O3—C21	1.384 (5)	C10—H10A	0.9900
O3—H3A	0.8501	C10—H10B	0.9900
N1—C2	1.315 (4)	C12—C13	1.387 (5)
N1—C3	1.389 (4)	C12—C17	1.401 (4)
N2—C2	1.351 (4)	C13—C14	1.380 (5)
N2—C8	1.388 (4)	C13—H13A	0.9500
N2—C9	1.470 (4)	C14—C15	1.392 (5)
N3—C11	1.317 (4)	C14—H14A	0.9500
N3—C12	1.397 (4)	C15—C16	1.378 (5)
N4—C11	1.345 (4)	C15—H15A	0.9500
N4—C17	1.378 (4)	C16—C17	1.382 (5)
N4—C18	1.457 (4)	C16—H16A	0.9500
N5—C19	1.155 (4)	C18—H18A	0.9800
N6—C20	1.154 (4)	C18—H18B	0.9800
C1—C2	1.498 (5)	C18—H18C	0.9800
C1—H1A	0.9900	C19—S1	1.636 (4)
C1—H1B	0.9900	C20—S2	1.630 (3)
C3—C4	1.388 (5)	C21—H21B	0.9800
C3—C8	1.405 (4)	C21—H21A	0.9800
C4—C5	1.384 (5)	C21—H21C	0.9800
C4—H4A	0.9500		
			
N6—Co1—N5	95.76 (12)	C7—C6—H6B	118.9
N6—Co1—N1	102.52 (11)	C5—C6—H6B	118.9
N5—Co1—N1	102.62 (10)	C6—C7—C8	116.5 (3)
N6—Co1—N3	96.87 (11)	C6—C7—H7A	121.7
N5—Co1—N3	101.37 (10)	C8—C7—H7A	121.7
N1—Co1—N3	147.26 (10)	N2—C8—C7	132.0 (3)
N6—Co1—O1	178.26 (10)	N2—C8—C3	105.7 (3)
N5—Co1—O1	84.12 (10)	C7—C8—C3	122.2 (3)
N1—Co1—O1	75.83 (9)	N2—C9—H9A	109.5
N3—Co1—O1	84.85 (9)	N2—C9—H9B	109.5
N6—Co1—O2	88.91 (11)	H9A—C9—H9B	109.5
N5—Co1—O2	173.52 (10)	N2—C9—H9C	109.5
N1—Co1—O2	80.65 (9)	H9A—C9—H9C	109.5
N3—Co1—O2	73.54 (9)	H9B—C9—H9C	109.5
O1—Co1—O2	91.34 (8)	O2—C10—C11	107.8 (3)
C1—O1—Co1	113.38 (19)	O2—C10—H10A	110.1
C1—O1—H1	116.7	C11—C10—H10A	110.1
Co1—O1—H1	100.9	O2—C10—H10B	110.1
C10—O2—Co1	114.71 (19)	C11—C10—H10B	110.1
C10—O2—H2A	106.8	H10A—C10—H10B	108.5
Co1—O2—H2A	116.5	N3—C11—N4	113.3 (3)
C21—O3—H3A	109.5	N3—C11—C10	123.5 (3)
C2—N1—C3	106.1 (3)	N4—C11—C10	123.2 (3)
C2—N1—Co1	117.9 (2)	C13—C12—N3	131.3 (3)
C3—N1—Co1	135.7 (2)	C13—C12—C17	119.5 (3)
C2—N2—C8	106.9 (3)	N3—C12—C17	109.2 (3)
C2—N2—C9	127.6 (3)	C14—C13—C12	117.4 (3)
C8—N2—C9	125.5 (3)	C14—C13—H13A	121.3
C11—N3—C12	104.8 (3)	C12—C13—H13A	121.3
C11—N3—Co1	118.5 (2)	C13—C14—C15	122.4 (4)
C12—N3—Co1	136.2 (2)	C13—C14—H14A	118.8
C11—N4—C17	107.4 (3)	C15—C14—H14A	118.8
C11—N4—C18	126.4 (3)	C16—C15—C14	121.0 (3)
C17—N4—C18	126.2 (3)	C16—C15—H15A	119.5
C19—N5—Co1	167.6 (3)	C14—C15—H15A	119.5
C20—N6—Co1	160.2 (3)	C15—C16—C17	116.6 (3)
O1—C1—C2	108.7 (3)	C15—C16—H16A	121.7
O1—C1—H1A	110.0	C17—C16—H16A	121.7
C2—C1—H1A	110.0	N4—C17—C16	131.6 (3)
O1—C1—H1B	110.0	N4—C17—C12	105.3 (3)
C2—C1—H1B	110.0	C16—C17—C12	123.2 (3)
H1A—C1—H1B	108.3	N4—C18—H18A	109.5
N1—C2—N2	112.9 (3)	N4—C18—H18B	109.5
N1—C2—C1	122.2 (3)	H18A—C18—H18B	109.5
N2—C2—C1	124.9 (3)	N4—C18—H18C	109.5
C4—C3—N1	131.5 (3)	H18A—C18—H18C	109.5
C4—C3—C8	120.0 (3)	H18B—C18—H18C	109.5
N1—C3—C8	108.5 (3)	N5—C19—S1	177.8 (3)
C5—C4—C3	117.7 (3)	N6—C20—S2	178.9 (3)
C5—C4—H4A	121.2	O3—C21—H21B	109.5
C3—C4—H4A	121.2	O3—C21—H21A	109.5
C4—C5—C6	121.3 (3)	H21B—C21—H21A	109.5
C4—C5—H5A	119.3	O3—C21—H21C	109.5
C6—C5—H5A	119.3	H21B—C21—H21C	109.5
C7—C6—C5	122.2 (3)	H21A—C21—H21C	109.5

### Hydrogen-bond geometry (Å, °)

**Table:**

*D*—H···*A*	*D*—H	H···*A*	*D*···*A*	*D*—H···*A*
O2—H2A···O3	0.85	1.89	2.689 (3)	155
O3—H3A···S2^i^	0.85	2.45	3.297 (3)	179
O1—H1···S1^i^	0.85	2.36	3.177 (2)	162

Symmetry codes: (i) *x*−1, *y*, *z*.
